# From Pixels to Principles: A Decade of Progress and Landscape in Trustworthy Computer Vision

**DOI:** 10.1007/s11948-024-00480-6

**Published:** 2024-06-10

**Authors:** Kexin Huang, Yan Teng, Yang Chen, Yingchun Wang

**Affiliations:** 1 Shanghai Artificial Intelligence Laboratory, Shanghai, 200232 China; 2 Fudan University, Shanghai, 200438 China

**Keywords:** Trustworthy AI, Computer vision, AI principles, Ethical AI, Progress and landscape

## Abstract

The rapid development of computer vision technologies and applications has brought forth a range of social and ethical challenges. Due to the unique characteristics of visual technology in terms of data modalities and application scenarios, computer vision poses specific ethical issues. However, the majority of existing literature either addresses artificial intelligence as a whole or pays particular attention to natural language processing, leaving a gap in specialized research on ethical issues and systematic solutions in the field of computer vision. This paper utilizes bibliometrics and text-mining techniques to quantitatively analyze papers from prominent academic conferences in computer vision over the past decade. It first reveals the developing trends and specific distribution of attention regarding trustworthy aspects in the computer vision field, as well as the inherent connections between ethical dimensions and different stages of visual model development. A life-cycle framework regarding trustworthy computer vision is then presented by making the relevant trustworthy issues, the operation pipeline of AI models, and viable technical solutions interconnected, providing researchers and policymakers with references and guidance for achieving trustworthy CV. Finally, it discusses particular motivations for conducting trustworthy practices and underscores the consistency and ambivalence among various trustworthy principles and technical attributes.

## Introduction

Computer Vision (CV) stands out as a pivotal field within artificial intelligence (AI), striving to impart machines with the ability to “perceive the world” akin to human visual cognition. This empowers computers to extract, analyze, and comprehend information from images and videos (Voulodimos et al., 2018). Within the realm of computer vision, commonplace undertakings encompass facial recognition, object detection, and action identification. Its significance is underscored by its widespread integration into our daily routines, such as identity verification at transportation hubs, facial payment systems, and cashier-less stores.

In recent years, the societal and ethical impacts of AI in general have come under scrutiny, and language models, such as ChatGPT and LLaMA, play an overwhelming role in the discourse. Nonetheless, the swift advancement of CV technology and its applications has ushered in an array of trustworthy dilemmas that are distinctive to this domain. Existing literature is missing a comprehensive analysis of ethical issues specific to computer vision tasks and applications.

Features that make the trustworthiness of CV unique are primarily three. The first and the most fundamental one refers to the unique manner in which information is conveyed within vision, mainly through images and videos. Unlike text data which usually contains rich semantic information, image data (e.g., object location, image content, etc.) can only show the information presented in the image while lacking information about the context, intentions, relationship, explicit grammatical rules, and structures. These intrinsic limitations of image data can result in ambiguities and potential misinterpretations, particularly when tasked with decision-making or predictions, which raises immediate concerns about the safety and potential misuse of CV systems.

Second, as raw image data often lacks information, the training process of CV models relies heavily on supervised learning and a large amount of human-labeled image data. However, the labeling process can be subjective and limited, which may amplify data bias regarding gender, age, ethnicity, skin color, etc. (Garcia, 2016; Buolamwini & Gebru, 2018; Raji & Buolamwini, 2019). Relatedly, as the performance of a CV model is highly dependent on the dataset used for training, the model may not be accurately generalized to new scenarios or tasks if the characteristics of the used dataset do not match the characteristics of the target application domain. Thus, these models often struggle to handle the complexity and diversity of real-world scenes, bearing poor robustness. The need for supervised learning can also make them vulnerable to adversarial attacks and leakage of training data, posing security and privacy concerns.

Finally, the tasks and application scenarios of CVs, such as medical imaging, automated driving, and security surveillance, are different from general AI systems. CV models need to accurately recognize images and videos and make appropriate predictions and decisions, requiring higher interpretability and transparency. Furthermore, these applications often encompass sensitive details such as facial features and medical records, thereby increasing potential threats to privacy, particularly considering that models have the capacity to deduce such information from medical images by reverse-engineering parameters (Shokri et al., 2017).

To provide a rich analysis of trustworthy issues concerning CV technology, this paper applies bibliometrics and text mining methods, then quantitatively and qualitatively analyzes the top conference literature in the CV field in the last 10 years. Based on careful review, a life-cycle trustworthiness framework is constructed by associating the different phases of the development of generalized computer vision models with specific trustworthy issues and solution measures.

## Related Work

### Trustworthy AI

It is a global consensus that establishing trustworthy AI is critical. In the past few years, there has been a surge in normative frameworks regarding trustworthy AI, ranging from ethical debates to specific constraints and restrictions such as policies, laws and regulations, and standards. In April 2019, the EU High-Level Expert Group on Artificial Intelligence formally released the *Ethics guidelines for trustworthy AI* (EU High-Level Expert Group on Artificial Intelligence, 2019), emphasizing that trustworthy AI should have the following three characteristics: (1) legal, i.e. complying with relevant legal requirements; (2) ethical, following ethical principles and values; and (3) robust, including both technical level and social environment level. Other similar documents include the *White Paper on Trustworthy Artificial Intelligence* published by Chinese Academy of Information and Communications Technology (Chinese Academy of Information and Communications Technology, 2023) and *Principles for the Stewardship of AI Applications* (USA) (Executive Office of the President Office of Management and Budget, 2020), among others, which show the global consensus on developing trustworthy AI (Jobin et al., 2019; Fjeld et al., 2020; Boza & Evgeniou, 2021; Vandemeulebroucke et al., 2022; Smallman, 2022; Liu et al., 2022).

While normative documents predominantly address AI in a broad context, it is acknowledged that certain elements are closely related to CV technologies. For instance, *the AI Act* (European Commission, 2021) mandates transparency in the creation and manipulation of image, audio, or video content that is significantly similar to genuine, real-world content. It also addresses specific transparency obligations regarding critical CV applications such as medical imaging and facial recognition. Such requirements underscore that existing literature and regulations offer valuable insights for addressing concerns specific to CV systems and provide a solid foundation for guidelines within specific CV domains. Despite this, there remains a notable gap in targeted research specifically for the domain of CV, which has led to the absence of potent regulation tailored to its unique challenges and potentialities. This paper aims to bridge this academic gap by reviewing the current landscape of solutions and proposing a trustworthy framework specifically designed for CV, building upon the foundational principles laid out in general AI discussions while addressing the distinct needs of CV applications.

### Bibliometrics

Bibliometrics is the study of documentary information, aiming to reveal trends and impacts of scientific research development through quantitative analysis utilizing statistical and measurement techniques (Broadus, 1987). Information like academic structures, corporations, and influences can be grasped under this approach and draw lessons for future study. For example, Zhang et al. (2021) explore the current status of research on ethics and privacy in the field of AI through bibliometric methods, providing a deeper insight to guide further research and policy-making. Meanwhile, Zhang et al. provide an empirical research demonstration for research methodology and application of bibliometrics in the field of AI, which guides the application of bibliometrics in this paper.

### Text Mining

Text mining is a process of automatically discovering, extracting, and deducing useful information from large-scale text data. It aims to uncover patterns, trends, and associations, providing insights and value to users (Hotho et al., 2005; Allahyari et al., 2017). In this paper, We utilize keyword extraction and topic modeling to filter and analyze articles based on the characteristics of the selected literature to overcome the manual research of a large value of selected literature.

#### Keyword Extraction

Keyword Extraction is an important task in text mining, aimed at automatically extracting the most representative and information-rich keywords or phrases from the text (Hulth, 2003). providing a foundation for tasks such as text understanding, retrieval, and classification. YAKE (Yet Another Keyword Extractor) (Campos et al., 2020; Campos et al., 2018a, 2018b) is an unsupervised keyword extraction algorithm that produces results with high accuracy and diversity, extracting multiple keywords relevant to different topics, It overcomes the limitation of traditional keyword extraction methods that often fail to capture key information expressed in phrases and multi-word expressions (Beliga, 2014; Hotho et al., 2005).

#### Topic Modeling

LDA (Latent Dirichlet Allocation) is a widely used probabilistic graphical model in the field of topic modeling. It’s used to discover hidden thematic structures within a dataset of text documents (Blei et al., 2003). It helps to understand the distribution of topics within large text datasets, allowing to discover key topics, track topic evolution, and explore relationships between topics (Chang et al., 2009; Yau et al., 2014; Kang et al., 2019). In this article, the LDA model is chosen to perform topic analysis on trustworthy relevant literature. The aim is to elucidate the relationship between the trustworthy dimension and computer technology and tasks, providing a better understanding of how visual models achieve trustworthiness.

## Method

**Fig. 1:**
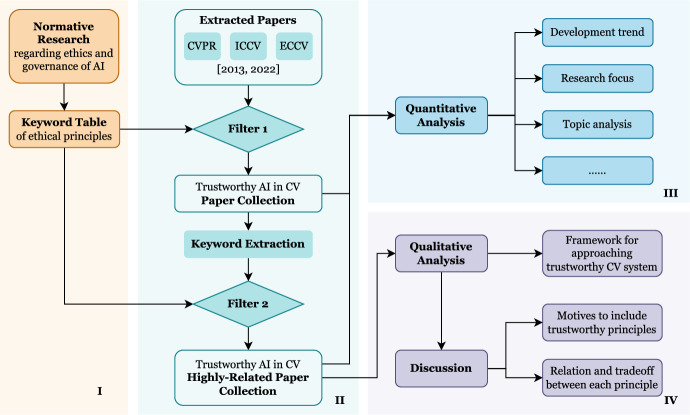
Overview of the paper

As shown in Fig. 1, we conduct our review in 4 steps:

- Part I: Design key principles. Primary principles and subcomponents are concluded by relative normative research.
- Part II: Literature filtering. Academic papers published at critical conferences in ten years are extracted and filtered twice for the most related results.
- Part III: Quantitative analysis. Filtered literature is analyzed to capture the embodiment of trustworthiness in the domain of CV.
- Part IV: Qualitative analysis. The filtered literature is qualitatively analyzed to construct a value framework.

### Keyword Table

Based on Jobin et al. (2019), Fjeld et al. (2020), Boza and Evgeniou (2021), Vandemeulebroucke et al. (2022), Smallman (2022), and Liu et al. (2022) in developing key dimensions of trustworthy AI, we conclude critical principles for trustworthy AI systems, as mentioned below:

- **Transparency** Trustworthy AI should be designed and implemented in a way that its operation can be monitored so that the results it produces are interpretable and transparent (Fjeld et al., 2020).
- **Fairness and Justice** Trustworthy AI should mitigate discrimination and bias toward any group of people throughout the life cycle of AI (Jobin et al., 2019).
- **Safety and Security** Trustworthy AI should avoid causing harm to humans (Kazim and Koshiyama, 2021). In this context, safety usually refers to the rational design and operation within the AI system, while security refers to the system’s ability to withstand attacks from outside (Jobin et al., 2019).
- **Responsibility** Involves a clear assignment of responsibility and auditability. It should be clear who should be held accountable for what impact of an AI system (Liu et al., 2022).
- **Privacy** Based on the need for privacy protection for the public and individuals, usually intertwined with data protection, data security and data governance (Andraško et al., 2021).
- **Freedom and Autonomy** Refers to the autonomy of the individual in his or her personal business, for example, AI system cannot collect and disseminate data on the user when people are unaware (Jobin et al., 2019).
- **Sustainability** The development of AI consumes much energy and should take into account environmental concerns. It also considers how AI could be used to improve the Earth’s ecosystems and biodiversity (Jobin et al., 2019).
- **Robustness** AI systems are required to be robust to perturbations and attacks in inputs and be able to generate safe outcomes (Liu et al., 2022).

### Literature Filter

#### Data Preprocessing

In this paper, we scrape open-access literature accepted by CVPR, ICCV, and ECCV over the past ten years, then filter twice to gain relative articles.

#### First Filtering

During the first filtering of literature, the subcomponents of each principle described in Sect. Keyword Table are processed, including adding synonyms and lexical variations to be maximally matched by regular expression. The final keyword list used for filtering is shown in Table 1.

**Table 1:** Keywords for filtering

Keyword table for filtering	
‘Transparency’, ‘transparent’, ‘explainability’, ‘explainable’, ‘explicable’, ‘audit’, ‘traceability’,	
‘Traceable’, ‘interpretability’, ‘interpretable’, ‘justice’, ‘fair’, ‘equality’, ‘discrimination’,	
‘Discriminate’, ‘prejudice’, ‘bias’, ‘stereotype’, ‘inclusion’, ‘inclusive’, ‘accessibility’, ‘accessible’,	
‘Impartial’, ‘safe’, ‘security’, ‘secure’, ‘prudence’, ‘precaution’, ‘non-maleficence’, ‘health’,	
‘Accountability’, ‘accountable’, ‘responsibility’, ‘responsible’, ‘integrity’, ‘responsive’, ‘privacy’,	
‘Private’, ‘data protection’, ‘data governance’, ‘data ownership’, ‘data quality’, ‘data integrity’,	
‘Dignity’, ‘confidential’, ‘consent’, ‘delegated’, ‘delegation’, ‘autonomy’, ‘freedom’,	
‘Human-control’, ‘sustainability’, ‘sustainable’, ‘well-being’, ‘peace’, ‘harmony’, ‘harmonious’,	
‘Resilience’, ‘resilient’, ‘robust’	

The trustworthy relevant literature that is finally included are articles meeting the following requirements (Filter 1):

- The title or abstract contains words from the keyword list
- The number of occurrences of the words in the keyword list in the full text is more than 20

The title and abstract are pivotal components of an article. Their inclusion of trustworthy terms suggests that the article places a significant emphasis on trustworthiness. Furthermore, in our experimentation with word occurrence thresholds of {5, 10, 15, 20, 25, 30}, a threshold of 20 proved superior. It effectively filters out unrelated articles while retaining the most pertinent ones. If an article contains more than 20 occurrences of words from our keyword list, it signifies that trustworthiness is not merely touched upon, but rather central to the discussion. This criterion helps exclude articles with only tangential relevance to trustworthiness.

#### Second Filtering

The YAKE keyword extraction technique is utilized in the second filtering The filtering rules are as follows:

- Use the YAKE keyword extractor to extract keywords from the first filtered articles (Filter 1) and output the 20 most relevant keywords per se.
- For the 20 extracted keywords in each article, if any of them are incorporated in Table 1, the article is considered to be highly relevant and will be included in the highly relevant literature pool.

Table 2 shows the one extracted results (Jung et al., 2022) ultimately labeled as highly relevant literature. This article proposes a new method for training fair classifiers with partially labeled group tags (like gender and race). As shown in the table, the keywords extracted by YAKE can validate the effectiveness of the second filtering.

**Table 2:** Example of keyword extraction by YAKE

Rank	Keyword	Score ($\downarrow$)	Rank	Keyword	Score ($\downarrow$)
1	Group labels	0.0006	11	Label	0.0043
2	Group	0.0011	12	Confidence-based group label	0.0051
3	Group **fairness**	0.0015	13	cgl	0.0053
4	Annotated group labels	0.0016	14	Pseudo group labels	0.0056
5	Labels	0.0027	15	Methods	0.0058
6	Group label assignment	0.0029	16	Training	0.0068
7	**Fairness**	0.0033	17	Random label	0.0068
8	Group classifier	0.0033	18	Annotated group	0.0073
9	Random group labels	0.0034	19	Group label regime	0.0075
10	Low group label	0.0035	20	Samples	0.0084

### Topic Modeling

LDA (Latent Dirichlet Allocation) is a generative model that treats documents as probability distributions over topics, each topic being a probability distribution over vocabulary (Blei et al., 2003). In this work, the title and abstract are selected as the data sources for topic modeling regarding the selection of data sources. Since the title and abstract often introduce the main ideas and contributions in a concise manner, therefore, they can be used for topic modeling to eliminate irrelevant information.

## Analysis

In this section, we quantitatively and qualitatively analyze our filtered literature.

### Quantitative Analysis

#### Overall Progress

The field of computer vision has been booming in recent years, producing a large number of milestone technical achievements, and the number of papers accepted at important conferences has been increasing yearly. CVPR accepted exceeded 2000 relevant articles for the first time in 2022, which is an increase of 24.11% compared with 1,663 papers in 2021.

This part counts the total number of papers accepted in the selected top conferences in ten years, as well as the number of trustworthy related articles and the increasing number of relevant papers each year. The total number of papers included in the three conferences in ten years is 19,375, including 1,162 trustworthy related articles and 533 highly related articles.

**Fig. 2:**
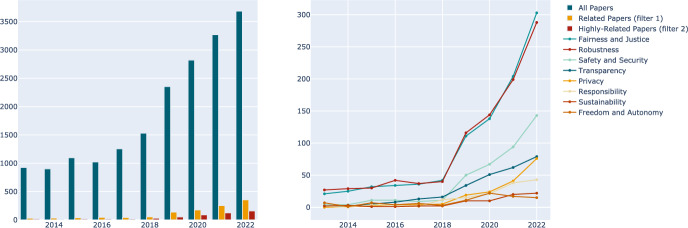
The evolution of the emergence of value norms in the literature (left) and statistics on the number of occurrences of each dimension’s value norms in the literature (right)

As shown in Fig. 2 (left), the total number of papers accepted in the three top conferences is demarcated by 2018, with a flat increase before 2019; and a rapid increase from 2019 to 2022. (Note: ICCV and ECCV are held in alternate years, and thus there may be a decrease in the number of articles in neighboring years.)

Also, we count the number of trustworthy related articles in each dimension over the decade. The statistical rule of this part is: if the paper is trustworthy relative (Filter 1), as long as the number of occurrences of the keyword in a dimension is not zero, it can be added to the according dimension.

The statistical results are shown in Fig. 2 (right). In general, the number of trustworthy related articles increased in most dimensions. Fairness and robustness-related articles have been the articles with the highest number of concerns; and their growth has accelerated even more after 2018, almost matching the growth trend of the total number of accepted papers. Security-related articles have grown sharply from almost zero after 2018, meanwhile, transparency and privacy-related articles have increased but at a slow pace. It is worth mentioning that in the years 2018 and 2019, many normative documents began to mention trustworthy AI worldwide, such as *Ethics guidelines for trustworthy AI* (EU High-Level Expert Group on Artificial Intelligence, 2019) and *Governance Principles for the New Generation Artificial Intelligence* (Ministry of Science and Technology of the People’s Republic of China, 2019). Both documents emphasized dimensions of human-centered, fair, safe, robust, explainable, and sustainable, which correspond to other relevant documents.

#### Distribution

**Fig. 3:**
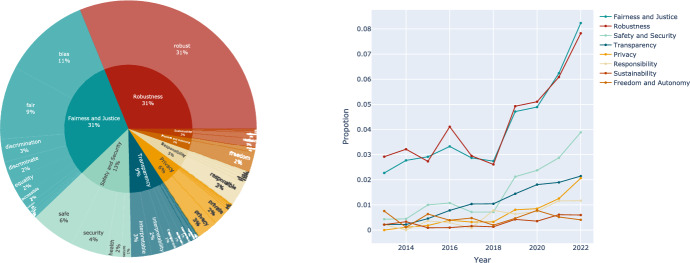
Statistics on the proportion of occurrences of each dimension’s value norms in the literature (left) and trends in the proportion of each dimension’s value norms appearing in the literature (right)

In this section, we focus on distributions of dimensions in filtered articles. Figure 3 (left) illustrates the overall distribution of dimensions over the decade, while the right subplot shows the increasing trend of each dimension.

It can be seen that in the field of CV, the attention paid to each dimension is uneven, robustness and fairness were emphasized most. Robustness in CV emphasizes the system’s capability to function effectively amidst the complexities and uncertainties of real-world environments. Unlike other dimensions, robustness is an intertwined indicator that has a technical aspect as well as an ethical aspect, and it overlaps safety and security in that CV models should be resilient to disturbances (e.g., Gaussian noise, impulse noise) and adversarial attacks (Hendrycks & Dietterich, 2019; Hendrycks et al., 2022).

Computer systems are increasingly utilized for making decisions that impact humans deeply, and an unfair system will lead to discrimination against subgroups of the population to a greater or lesser extent (Friedler et al., 2019). Models may have increased unfairness due to their opacity, training with biased data, and being sensitive to certain metrics. For example, CV technologies are used in crime prediction and policing applications. However, these systems can suffer from over-monitoring and conviction bias against specific groups, leading to unfair enforcement and sentencing. Likewise, In medical imaging diagnostic applications, such as breast cancer detection and skin disease diagnosis, computer vision models may have variations in diagnostic accuracy between genders and races, leading to medical misdiagnosis or underdiagnosis of certain groups (Obermeyer et al., 2019).

As Fig. 3 (right) shows, safety and security have also received greater attention since 2018. The emergence of computer vision applications emphasizes the need for reliable internal systems to resist external attacks. For example, the internal part of for self-driving system needs to accurately recognize pedestrians, traffic lights, obstacles, etc., in order to safeguard the safety of the occupants and pedestrians on the road, etc. (Hussain & Zeadally, 2019). At the same time, it needs to have the ability to resist external attacks and perform correct judgment and decision-making when an attacker tries to, for example, modify a stop sign through a small perturbation (Eykholt et al., 2018).

As deep neural networks are widely used in visual modeling and their internal structure is complex, it is difficult to attribute their prediction. Researchers have been working to improve the interpretability of models, such as visualizing and classing activation maps (Zhou et al., 2015; Simonyan et al., 2014), analyzing gradient information (Selvaraju et al., 2017), and interpretable GANs (Bau et al., 2018). For privacy, there are significant risks in terms of data collection, storage, processing, and sharing. Advancements in the law and increased social awareness inevitably require AI to respect individual privacy.

Apart from the uneven distribution of each principle, we observe that there is a significant difference between the value requirements for general AI and the domain of CV. Table 3 selects the ethical principles summarized by Jobin et al. as well as the top 5 trustworthy CV principles summarized by this paper.

**Table 3:** Differences between AI generic value specification requirements and extant value concerns in computer vision

General AI	Percentage in nor-mative documents	CV	Attention distribution
Transparency	86.9%	Robustness	31.2%
Justice and fairness	80.9%	Fairness and Justice	31.0%
Non-maleficence	71.4%	Safety and Security	13.2%
Responsibility	71.4%	Transparency	8.9%
Privacy	55.9%	Privacy	5.76%

When comparing the value concerns between the general field of AI and CV, certain differences emerge. Transparency stands out as a predominant concern in the broader realm of AI, with 86.9% attention in normative documents, underscoring the importance of understanding how AI systems arrive at decisions for reviewing and validation purposes. In comparison, transparency receives 8.9% attention in CV technical literature relevant to trustworthiness. This figure is relatively low because it is vital to decipher decision-making processes in CV systems. Fairness takes second place on both general AI and CV, showing the consistency of attention received by this principle. Emphasized at 71.4%, non-maleficence highlights harm prevention physically or psychologically. This concern parallels the CV’s emphasis on safety and security at 13.2%, focusing on shielding CV systems from malicious attacks and misuse. Meanwhile, while Responsibility reflected 71.4% of normative documents, it is out of the top 5 concerns of CV-relevant research. Nevertheless, this does not mean that the attribution of responsibility is not important in CV systems. It merely indicates that technical solutions might not be the commonest way to tackle this issue compared to laws, regulations, and management mechanisms. Likewise, privacy takes the same place on both sides, with 55.9% attention in general AI and 5.76% attention in CV, emphasizing the importance of personal data protection in visual datasets and outputs. As mentioned, robustness receives the highest attention in relevant CV research as it is not merely an important intertwined indicator but also overlaps safety and security. Notably, while robustness is not on the list of ethical principles of general AI, this does not mean that it is not important. As explained in Sect. Trustworthy AI, robustness is often regarded as part of trustworthy AI, which is in parallel with ethical AI, This might explain why Jobin et al. do not take robustness into consideration.

#### Topic Modeling Result

**Fig. 4:**
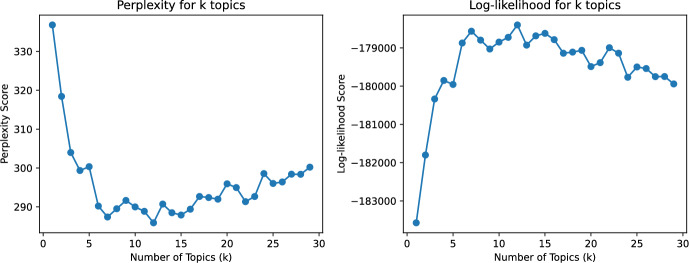
LDA topic modeling on trustworthy related papers (Filter 2)

We do LDA modeling with hyperparameters of topic numbers [2, 30] among trustworthy highly related papers (Filter 2), the result is displayed in Fig. 4.

It can be seen that for the perplexity ($\downarrow {}$) index, there is a trend from decreasing to increasing, indicating that there is an optimal number of topics that can classify trustworthiness-related literature; the similar trend embodied in log-likelihood value ($\uparrow {}$) as well, validating the credibility the optimal number of topics. By manually reviewing the topic analysis results of two perplexity and log-likelihood values that perform better for the number of topics k = 7 and k = 12, we found that the classification of documents is slightly repeated and many topics can be further combined when the number of topics k = 12. Therefore, we selected the number of topics k = 7 for subsequent analysis.

**Table 4:**
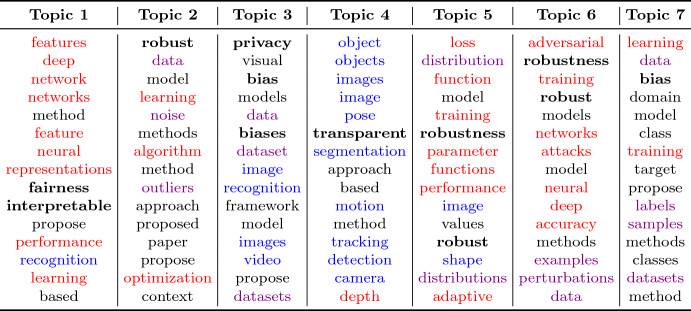
Topic modeling result on trustworthy highly-related papers ($\hbox {k} = 7$)

Table 4 shows the 7 topics of LDA topic modeling on the trustworthy highly-related literature and the top 15 results of each topic which are labeled as follows:

- The keywords appearing in the keyword table (Table 1) are **bold**. For example, “interpretable”, “privacy”, etc.
- Keywords related to “model” are highlighted in red. For example, “deep”, “training”, “optimization”, and “parameter”, etc.
- Keywords related to “data” are highlighted in violet. For example, “data”, “outliers”, and “labels”.
- Keywords related to “task” are highlighted in blue. For example, “recognition”, “tracking” and “segmentation”, etc.
- Keywords that have no obvious meaning in the CV field have not been specifically labeled, such as “propose”, or “based”; and words that appear in almost every article, such as “method”, “model”, etc.

We can see some intriguing topic modeling results. First, almost all dimensions are grouped under different themes. Second, under different topics, the distribution of dimensions related to “model” / “data” / “task” is very distinctive.

The distribution of “robust” and “robustness” is most distinctive. “Robustness” does not appear with any other dimension under the same topic, but is strongly related to the “model” (Topics 5 and 6) itself. Deep learning technology suffers from risks of low explainability and uncertainty, and is prone to produce incorrect or unexpected outputs when facing adversarial threats. Thus, researchers endeavor to improve the robustness of the model for withstanding various attacks (Topic 5) and maintaining high accuracy on attacks against adversarial samples (Topic 6). Additionally, Topic 2 demonstrates that the model needs robustness against noise or outliers, etc.

In Topic 1, “fairness” and “interpretable” both appear, with many keywords attached to “model” altogether. In many cases, “interpretable” is seen as the precondition of “fairness”. The comprehension of the reasoning of CV models, which is referred largely to as representations that transform the raw textual data into a machine-understandable format and are used for processing and understanding textual data within the model, is the key to ensuring the fairness of the generated result. In other words, transparency often relates to the representations inside the model, if the model’s representations are clear and interpretable, then the model’s transparency is likely higher because people can more easily understand how the model processes data and makes decisions. Consequently, it would be easier to detect and improve fairness issues.

Bias appears in both Topic 3 and Topic 7, with the appearance of a large number of “data” related keywords. Since data is the foundation for training models, the relative deviations will impact the results of the model. For illustration, in terms of the image classification task, if the training data set is occupied with male images, the model may result in poor performance when identifying women. Therefore, when building trustworthy CV models, special attention needs to be paid to data collection and annotation to ensure that the data set achieves equilibrium of samples of genders, races, cultures, and economic backgrounds to reduce bias. Meanwhile, the keywords “privacy” and “bias” both appear in Topic 3, probably because the training data of CV models often contains users’ sensitive information. While issues of privacy must be considered when processing data, it should be noted that measures such as data desensitization and differential privacy can lead to certain processing and distortion of the data, which may also lead to bias.

Topic 4 highlights the correlation between “transparent” and specific “tasks” that users are incapable of understanding the model behavior without explanations. For example, tracking tasks involve tracking the position and movement of objects in video sequences. Transparency can reveal how the model recognizes and tracks objects, and how the model handles occlusions, lighting changes, and other complex situations. Likewise, the pose estimation task involves estimating and inferring the pose of a human body or object. Transparency can reveal how the model analyzes key points and structures in the image to infer further gesture information, which aids users in understanding the reasoning behind the model.

### Qualitative Analysis: A Life-Cycle Trustworthiness Framework for Frontier CV Models

Apart from quantitative analysis, we manually analyzed 272 CVPR papers in a highly related literature pool to explore detailed patterns.

**Fig. 5:**
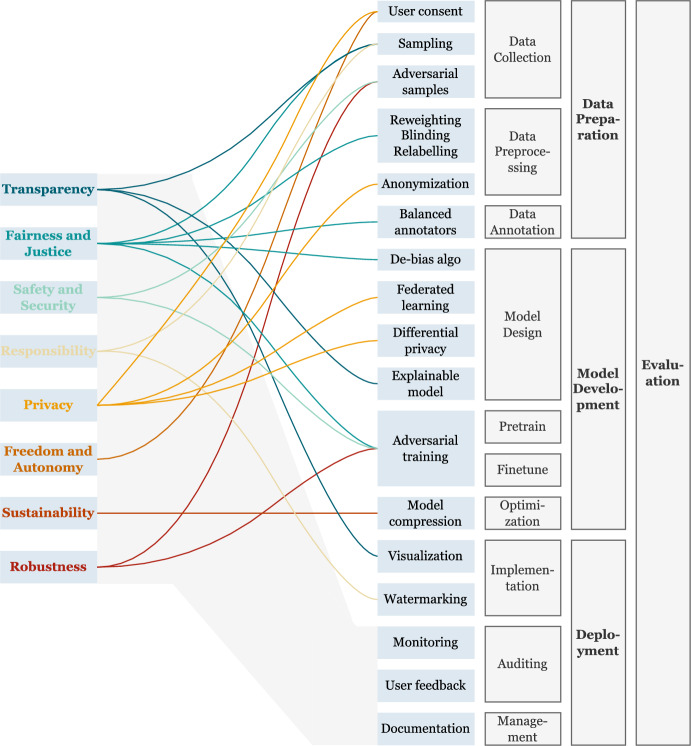
Life-cycle trustworthiness framework of frontier CV models

In recent years, there has been a global consensus on developing trustworthy and responsible AI (Bommasani et al., 2021) and bridging the gap between theories and practices (Li et al., 2023; Bleher & Braun, 2023). Among them, the construction of an interdisciplinary and systematic AI governance framework has received increasing attention. In the following section, we (1) summarize the previous data and qualitative analysis results from the perspectives of different stages of “frontier CV models” (referred to as foundation models that can be applied to a wide variety of tasks), and (2) provide targeted measures for trustworthy AI at different stages of general CV, to enhance the alignment between general CV models and ethical standards, laws and regulations (Fig. 5).

In the results of the topic modeling, it can be seen that attention toward different dimensions of trustworthiness is scattered in various aspects including “data”, “model” and “task”, providing insights and guidance for summarizing a trustworthy framework.

In the **data collection** stage, the most relevant dimensions are fairness, privacy, robustness, and autonomy. Data owners shall be informed of protocols regarding data collection, storage, and process. And data could not be processed without the consent given by the data owners. Moreover, the prevention of data leakage must be implemented. When building datasets, we should (1) be prudent of data labeling when encountering sensitive personal information, (2) lecture annotators with sufficient knowledge in identifying personal information and sensitive information, (3) ensure the diversity of annotators, and (4) ensure training data are diverse and fair which includes the equitable representation of races, ages and genders to promote similar performance across groups (Zhang et al., 2017). The robustness of the model can be evaluated by adding noise and perturbation to the large-scale image dataset to construct an adversarial sample set (Hendrycks et al., 2021), providing more complete head pose variations in the face dataset, or reducing the image quality (Klare et al., 2015).

During the **model development** phase, issues around robustness, privacy, fairness, interpretability, and sustainability are more severe. Techniques like adversarial training and robust optimization can enhance a model’s robustness. Privacy of raw data can be protected through mechanisms including federated learning and differential privacy while limiting the memorization of private details. Fairness may be promoted by reweighting, regularization, and timely human supervision to align with human values during training (Liu et al., 2022). Interpretable models and algorithms make the decision-making process more transparent by utilizing decision trees, rule models, or attention mechanisms. Visualization tools can also illustrate a model’s focus points and reasoning on input data. Regarding sustainability, energy efficiency measures during training are encouraged to optimize resource consumption and reduce carbon emissions.

During the **deployment** stage, usually including finetuning and application, considerations of trustworthiness become more complex as models interact closely with reality and society. Technically, robust and reliable systems require resilience against malicious attacks without compromising safety. Privacy necessitates dataset protections and the preclusion of overly realistic outputs. Fairness demands representation equity in training data. Regulatory requirements include transparency through detectability, interpretability, and ongoing oversight. Meanwhile, relevant agencies should deploy policies to prevent misuse and the generation of false and deceptive or falsified images. And there is a need to set accountability mechanisms in case CV models make poor decisions that lead to serious consequences.

Meanwhile, the **evaluation** process should be incorporated in three stages of data deployment, model training, finetuning, and application deployment. For data preparation, the reliability, accuracy, and relevance of data sources need to be evaluated to ensure the dataset is constructed based on trustworthy sources. Data quality assessment including consistency, noise, missing values, and distribution of the data is necessary as well to mitigate adverse impacts. In the model development phases, not only do we need comparison with baseline methods or human experts which are required to assess model performance, but we also need to assess the robustness regarding disturbances, noise, and adversarial attacks, to ensure reliable practical applications. After deployment, the integration and compatibility of the model should be evaluated to ensure the function. Additionally, the assessment of feedback should be introduced to optimize the model’s performance and user experience. Relevant institutions should also conduct periodic reviews on signs of misuse, safety incidents, and major deployment cases.

## Discussion

Following quantitative and qualitative analysis, we discuss specific motives and highlight the consistencies and contradictions among different trustworthy dimensions and technical indicators.

### Motives to Consider Trustworthy Principles

First, the dimensions of security, privacy, and responsibility carry significant implications for the stability of society, and therefore the development of technology must adherent to relevant laws and regulations. Owing to CV requiring the processing of personal data, researchers must avoid the possibility of data leakage in order to protect privacy and comply with legal requirements such as the EU’s GDPR and the California Consumer Privacy Act (Luo et al., 2021). Following these requirements, Zhu et al. (2022) argue that facial image data from the public should not be uploaded to the cluster and identity-related annotations should not be provided. Dusmanu et al. (2021) focus on concerns about inferring private information by reverse recovery, and propose a privacy-preserving feature representation that makes it difficult for an attacker to infer original information.

Second, research on robustness, security, and interpretability is principal to the ameliorating of model performance. As an illustration, the interpretability of deep neural networks is crucial for clinical applications in digital pathology, where today’s popular pixelation-based processing techniques ignore biological entity concepts, making it difficult for human experts to understand model results. Jaume et al. (2021) propose new quantitative metrics that use pathologically measurable concepts to characterize the graphical interpreter to explain the CytoMAP representations of breast cancer subtypes and the metrics can be generalized to other domains as well.

Third, implementing dimensions such as transparency, fairness, sustainability and accountability into CV practices can accelerate social benefits and social acceptance of new technologies (Kieslich et al., 2022). For example, recruitment systems should not set different probabilities of recommendation because candidates are from different demographic groups, and the introduction of risk assessment systems could eliminate racism (Wang et al., 2022). Likewise, Beery et al. (2022) focused on the specific task of urban forest detection and endeavored to advance computer vision systems to solve real-world problems to contribute to sustainable development and human well-being.

In recent years, academic literature has begun to focus exclusively on value specification. For example, Hirota et al. (2022) focused on social bias under the image captioning task. Zhang et al. (2017) proposed UTKFace, a dataset balanced in terms of gender and age, aiming to ameliorate the accuracy of face recognition systems for different demographic subgroups. Besides, many technical papers have started to mention dimensions such as privacy and unfairness dimensions and generalized trustworthy AI issues in the “Limitation/Discussion/Societal Impact” section at the end of the article.

### Intertwined Relations Between Principles

Principles do not exist in isolation; instead, they exhibit coherence and contradiction across various dependable dimensions. Take into account the coherence, where enhancing the effectiveness of one dimension will yield positive impacts on other dimensions as well. There is a consistent relationship between robustness and security. For example, regarding self-driving, the visual model needs to have resistance to perturbations that exist in reality, such as blizzard weather, damaged road signs, etc. (Eykholt et al., 2018), which is also a reflection of security. Furthermore, enhancing the interpretability of models aids developers and users in understanding the decision-making process of the model, making it easier to identify and correct potential biases, thereby increasing the algorithm’s fairness (Quadrianto et al., 2019). Meanwhile, methods to prevent the reconstruction of original images from visual descriptors can improve privacy as well as increase the security of data processing (Ng et al., 2022).

Besides, there may be ambivalence between different dimensions such as privacy and interpretability. According to Zhao et al. (2021), Explainable Artificial Intelligence (XAI) provides more information to improve explainability, which can inadvertently reveal sensitive information about the input data. This information can be used by attackers to reconstruct the original data, which may include sensitive or private information. This contradiction calls for attention to new privacy-preserving techniques that lead to an equilibrium between explainability and privacy. Another illustration is fairness and accuracy. Facial recognition systems often pursue high accuracy rates while ignoring differences in performance across population subgroups, while systems that prioritize fairness may lack accuracy (Wang & Deng, 2020). Robustness and accuracy are also in conflict. For example, defense strategies in adversarial research, like projecting images onto the image manifold, may bias the system towards database image distributions, causing misclassifications of clean images not well represented. This highlights a prevalent trade-off, where increasing robustness to attacks can reduce accuracy on unaltered data, and vice versa (Dubey et al., 2019).

## Conclusion

This paper has closely engaged with the ethical and social aspects of CV by examining relevant articles accepted in three major CV conferences in recent ten years, providing a first, comprehensive analysis and framework regarding trustworthy CV.

Based on the quantitative results, we observe that the attention to trustworthiness displays a rising trend after 2018, with the global consensus on building trustworthy AI. While the current distribution of dimensions is not balanced, Fairness and robustness are most emphasized. From the results of topic modeling, dimensions distributed differently related to aspects of vision models (“model”, “data”, “task”). For instance, robustness is more related to the performance of the model and the training method, which requires the model to be able to cope with adversarial attacks and make correct decisions when facing noise and outliers. Fairness and privacy are more related to the data. Transparency is often co-occurring with “task”, emphasizing the common need for transparency in various visual tasks.

Also, we conduct qualitative analysis for the subset of collected literature and provide a life-cycle framework regarding trustworthy CV. We summarize the current feasible solutions from the trustworthy problems in the three phases of data preparation, model development, and deployment, providing references and suggestions for researchers and policymakers to establish trustworthy visual models. Furthermore, we discuss the main motives of dimensions in the literature on CV, including compliance with legal requirements, enhancement of social benefits, and ameliorating models’ performance. Additionally, it should be noted that coherence and contradictions exist between dimensions and technical indicators, and researchers, policy-makers, and the public should be aware and need to weigh the specific situation in order to maximize the interests of human beings.

## References

[CR1] Allahyari, M., Pouriyeh, S., Assefi, M., Safaei, S., Trippe, E. D., Gutierrez, J. B., & Kochut, K. (2017). A brief survey of text mining: Classification, clustering and extraction techniques.

[CR2] Andraško, J., Mesarčík, M., & Hamul’ák, O. (2021). The regulatory intersections between artificial intelligence, data protection and cyber security: Challenges and opportunities for the EU legal framework. *AI and Society,* 1–14.

[CR3] Bau, D., Zhu, J.-Y., Strobelt, H., Zhou, B., Tenenbaum, J. B., Freeman, W. T., & Torralba, A. (2018). GAN dissection: Visualizing and understanding generative adversarial networks.

[CR4] Beery, S., Wu, G., Edwards, T., Pavetic, F., Majewski, B., Mukherjee, S., Chan, S., Morgan, J., Rathod, V., & Huang, J. (2022). The auto arborist dataset: A large-scale benchmark for multiview urban forest monitoring under domain shift. In *Proceedings of the IEEE/CVF conference on computer vision and pattern recognition* (pp. 21294–21307).

[CR5] Beliga, S. (2014). Keyword extraction: A review of methods and approaches. University of Rijeka, Department of Informatics, Rijeka 1(9).

[CR6] Bleher H, Braun M (2023). Reflections on putting AI ethics into practice: How three AI ethics approaches conceptualize theory and practice. Science and Engineering Ethics.

[CR7] Blei DM, Ng AY, Jordan MI (2003). Latent Dirichlet allocation. Journal of Machine Learning Research.

[CR8] Bommasani, R., Hudson, D. A., Adeli, E., Altman, R., Arora, S., Arx, S., Bernstein, M. S., Bohg, J., Bosselut, A., Brunskill, E., et al. (2021). On the opportunities and risks of foundation models. arXiv preprint arXiv:2108.07258

[CR9] Boza, P., & Evgeniou, T. (2021). Implementing AI principles: Frameworks, processes, and tools. INSEAD Working Paper No. 2021/04/DSC/TOM.

[CR10] Broadus RN (1987). Toward a definition of “bibliometrics”. Scientometrics.

[CR11] Buolamwini, J., & Gebru, T. (2018). Gender shades: Intersectional accuracy disparities in commercial gender classification. In *Conference on fairness, accountability and transparency* (pp. 77–91). PMLR.

[CR12] Campos, R., Mangaravite, V., Pasquali, A., Jorge, A. M., Nunes, C., Jatowt, A. (2018). A text feature based automatic keyword extraction method for single documents. In *Advances in information retrieval: 40th European conference on IR research (ECIR 2018)*, *Proceedings 40, *(pp. 684–691), Grenoble, France, March 26–29, 2018. Springer.

[CR13] Campos, R., Mangaravite, V., Pasquali, A., Jorge, A. M., Nunes, C., Jatowt, A. (2018). Yake! Collection-independent automatic keyword extractor. In *Advances in information retrieval: 40th European conference on IR research (ECIR 2018), Proceedings 40, *(pp. 806–810)*, *Grenoble, France, March 26–29, 2018. Springer.

[CR14] Campos R, Mangaravite V, Pasquali A, Jorge A, Nunes C, Jatowt A (2020). Yake! keyword extraction from single documents using multiple local features. Information Sciences.

[CR15] Chang, J., Gerrish, S., Wang, C., Boyd-Graber, J., & Blei, D. (2009). Reading tea leaves: How humans interpret topic models. *Advances in Neural Information Processing Systems, **22*.

[CR16] Chinese Academy of Information and Communications Technology. (2023). White paper on trustworthy artificial intelligence. http://www.caict.ac.cn/kxyj/qwfb/bps/202401/t20240122_470753.htm

[CR17] Dubey, A., Maaten, L.V.D., Yalniz, Z., Li, Y., & Mahajan, D. (2019). Defense against adversarial images using web-scale nearest-neighbor search. In *Proceedings of the IEEE/CVF conference on computer vision and pattern recognition* (pp. 8767–8776).

[CR18] Dusmanu, M., Schonberger, J. L., Sinha, S. N., & Pollefeys, M. (2021). Privacy-preserving image features via adversarial affine subspace embeddings. In *Proceedings of the IEEE/CVF conference on computer vision and pattern recognition* (pp. 14267–14277).

[CR19] EU High-Level Expert Group on Artificial Intelligence. (2019). Ethics guidelines for trustworthy AI. https://digital-strategy.ec.europa.eu/en/library/ethics-guidelines-trustworthy-ai

[CR20] European Commission. (2021). EU Artificial Intelligence Act. https://artificialintelligenceact.eu/the-act/

[CR21] Executive Office of the President Office of Management and Budget. (2020). Principles for the stewardship of AI applications.

[CR22] Eykholt, K., Evtimov, I., Fernandes, E., Li, B., Rahmati, A., Xiao, C., Prakash, A., Kohno, T., & Song, D. (2018). Robust physical-world attacks on deep learning models.

[CR23] Fjeld, J., Achten, N., Hilligoss, H., Nagy, A., & Srikumar, M. (2020). Principled artificial intelligence: Mapping consensus in ethical and rights-based approaches to principles for AI. Berkman Klein Center Research Publication (2020-1).

[CR24] Friedler, S. A., Scheidegger, C., Venkatasubramanian, S., Choudhary, S., Hamilton, E. P., Roth, D. (2019). A comparative study of fairness-enhancing interventions in machine learning. In *Proceedings of the conference on fairness, accountability, and transparency. FAT* ’19* (pp. 329–338). Association for Computing Machinery. 10.1145/3287560.3287589

[CR25] Garcia M (2016). Racist in the machine. World Policy Journal.

[CR26] Hendrycks, D., & Dietterich, T. (2019). Benchmarking neural network robustness to common corruptions and perturbations. arXiv preprint arXiv:1903.12261

[CR27] Hendrycks, D., Carlini, N., Schulman, J., & Steinhardt, J. (2022). Unsolved problems in ML safety.

[CR28] Hendrycks, D., Zhao, K., Basart, S., Steinhardt, J., & Song, D. (2021). Natural adversarial examples. In *Proceedings of the IEEE/CVF conference on computer vision and pattern recognition* (pp. 15262–15271).

[CR29] Hirota, Y., Nakashima, Y., & Garcia, N. (2022). Quantifying societal bias amplification in image captioning. In *Proceedings of the IEEE/CVF conference on computer vision and pattern recognition* (pp. 13450–13459).

[CR30] Hotho A, Nürnberger A, Paaß G (2005). A brief survey of text mining. Journal for Language Technology and Computational Linguistics.

[CR31] Hulth, A. (2003). Improved automatic keyword extraction given more linguistic knowledge. In *Proceedings of the 2003 conference on empirical methods in natural language processing* (pp. 216–223).

[CR32] Hussain R, Zeadally S (2019). Autonomous cars: Research results, issues, and future challenges. IEEE Communications Surveys and Tutorials.

[CR33] Jaume, G., Pati, P., Bozorgtabar, B., Foncubierta, A., Anniciello, A. M., Feroce, F., Rau, T., Thiran, J.-P., Gabrani, M., & Goksel, O. (2021) Quantifying explainers of graph neural networks in computational pathology. In *Proceedings of the IEEE/CVF conference on computer vision and pattern recognition* (pp. 8106–8116).

[CR34] Jobin A, Ienca M, Vayena E (2019). The global landscape of AI ethics guidelines. Nature Machine Intelligence.

[CR35] Jung, S., Chun, S., Moon, T. (2022). Learning fair classifiers with partially annotated group labels. In *Proceedings of the IEEE/CVF conference on computer vision and pattern recognition* (pp. 10348–10357).

[CR36] Kang HJ, Kim C, Kang K (2019). Analysis of the trends in biochemical research using latent Dirichlet allocation (LDA). Processes.

[CR37] Kazim E, Koshiyama AS (2021). A high-level overview of AI ethics. Patterns.

[CR38] Kieslich K, Keller B, Starke C (2022). Artificial intelligence ethics by design evaluating public perception on the importance of ethical design principles of artificial intelligence. Big Data and Society.

[CR39] Klare, B. F., Klein, B., Taborsky, E., Blanton, A., Cheney, J., Allen, K., Grother, P., Mah, A., Burge, M., & Jain, A. K. (2015). Pushing the frontiers of unconstrained face detection and recognition: Iarpa Janus benchmark A. In *2015 IEEE conference on computer vision and pattern recognition (CVPR)* (pp. 1931–1939). 10.1109/CVPR.2015.7298803

[CR61] Li Bo, Wu M, Tian GY, Zhang G, Lu J (2021). Trustworthy AI: From principles to practices. ACM Compututing Surveys.

[CR40] Liu H, Wang Y, Fan W, Liu X, Li Y, Jain S, Liu Y, Jain A, Tang J (2022). Trustworthy AI: A computational perspective. ACM Transactions on Intelligent Systems and Technology.

[CR41] Luo, Z., Wu, D. J., Adeli, E., & Fei-Fei, L. (2021). Scalable differential privacy with sparse network finetuning. In *Proceedings of the IEEE/CVF conference on computer vision and pattern recognition* (pp. 5059–5068).

[CR42] Ministry of Science and Technology of the People’s Republic of China. (2019). Governance Principles for the new generation artificial intelligence. https://www.most.gov.cn/kjbgz/201906/t20190617_147107.html

[CR43] Ng, T., Kim, H. J., Lee, V. T., DeTone, D., Yang, T. -Y., Shen, T., Ilg, E., Balntas, V., Mikolajczyk, K., & Sweeney, C. (2022). Ninjadesc: Content-concealing visual descriptors via adversarial learning. In *Proceedings of the IEEE/CVF conference on computer vision and pattern recognition* (pp. 12797–12807).

[CR44] Obermeyer Z, Powers B, Vogeli C, Mullainathan S (2019). Dissecting racial bias in an algorithm used to manage the health of populations. Science.

[CR45] Quadrianto, N., Sharmanska, V., & Thomas, O. (2019). Discovering fair representations in the data domain. In *Proceedings of the IEEE/CVF conference on computer vision and pattern recognition* (pp. 8227–8236).

[CR46] Raji, I. D., & Buolamwini, J. (2019). Actionable auditing: Investigating the impact of publicly naming biased performance results of commercial AI products. In *Proceedings of the 2019 AAAI/ACM conference on AI, ethics, and society* (pp. 429–435).

[CR47] Selvaraju, R. R., Cogswell, M., Das, A., Vedantam, R., Parikh, D., & Batra, D. (2017). Grad-cam: Visual explanations from deep networks via gradient-based localization. In *2017 IEEE international conference on computer vision (ICCV)* (pp. 618–626). 10.1109/ICCV.2017.74

[CR48] Shokri, R., Stronati, M., Song, C., & Shmatikov, V. (2017). Membership inference attacks against machine learning models.

[CR49] Simonyan, K., Vedaldi, A., & Zisserman, A. (2014). Deep inside convolutional networks: Visualising image classification models and saliency maps.

[CR50] Smallman M (2022). Multi scale ethics-why we need to consider the ethics of AI in healthcare at different scales. Science and Engineering Ethics.

[CR51] Vandemeulebroucke T, Denier Y, Mertens E, Gastmans C (2022). Which framework to use? A systematic review of ethical frameworks for the screening or evaluation of health technology innovations. Science and Engineering Ethics.

[CR52] Voulodimos, A., Doulamis, N., Doulamis, A., Protopapadakis, E., et al. (2018). Deep learning for computer vision: A brief review. In *Computational intelligence and neuroscience 2018*. 10.1155/2018/7068349 PMC5816885 29487619

[CR53] Wang, M., & Deng, W. (2020). Mitigating bias in face recognition using skewness-aware reinforcement learning. In *Proceedings of the IEEE/CVF conference on computer vision and pattern recognition (CVPR)*.

[CR54] Wang, Z., Dong, X., Xue, H., Zhang, Z., Chiu, W., Wei, T., & Ren, K. (2022). Fairness-aware adversarial perturbation towards bias mitigation for deployed deep models. In *Proceedings of the IEEE/CVF conference on computer vision and pattern recognition* (pp. 10379–10388).

[CR55] Yau C-K, Porter A, Newman N, Suominen A (2014). Clustering scientific documents with topic modeling. Scientometrics.

[CR56] Zhang, Z., Song, Y., & Qi, H. (2017). Age progression/regression by conditional adversarial autoencoder. In *Proceedings of the IEEE conference on computer vision and pattern recognition (CVPR)*.

[CR57] Zhang Y, Wu M, Tian GY, Zhang G, Lu J (2021). Ethics and privacy of artificial intelligence: Understandings from bibliometrics. Knowledge-Based Systems.

[CR58] Zhao, X., Zhang, W., Xiao, X., & Lim, B. (2021). Exploiting explanations for model inversion attacks. In *Proceedings of the IEEE/CVF international conference on computer vision (ICCV)* (pp. 682–692).

[CR59] Zhou, B., Khosla, A., Lapedriza, A., Oliva, A., & Torralba, A. (2015). Learning deep features for discriminative localization.

[CR60] Zhu, W., Wang, C.-Y., Tseng, K.-L., Lai, S.-H., & Wang, B. (2022). Local-adaptive face recognition via graph-based meta-clustering and regularized adaptation. In *Proceedings of the IEEE/CVF conference on computer vision and pattern recognition* (pp. 20301–20310).

